# 597 Reconstruction of Lop Ear Deformities After Severe Burn Injury in Pediatric Patients: An 11-year Experience

**DOI:** 10.1093/jbcr/irac012.225

**Published:** 2022-03-23

**Authors:** Richard J Ehrlichman, Farzin Sadeq, Daniel N Driscoll

**Affiliations:** MGH Northshore Medical Center, Boston, Massachusetts; Shriners Hospitals for Children Boston, Boston, Massachusetts; Massachusetts General Hospital, Boston, Massachusetts

## Abstract

**Introduction:**

The ear is one of the most highly visible structures on the face. One or both ears are frequently involved in panfacial burns. Because they are bilateral structures, asymmetries are clearly visible. Reconstruction of burned ear deformity often requires replacement or modification of skin and/or cartilage. Even these are successfully replaced, scar contractile forces can cause deformation of the ear. One of these deformities is lop or cup ear, in which the helical rim is either folded over, wrinkled or tight. We describe the necessity of applying otoplasty techniques to address this problem.

**Methods:**

A retrospective chart review analysis was performed to assess demographic variables, procedural indications and procedural outcomes in pediatric patients treated at our hospital for complex ear reconstruction for ear deformities after her surgery. Inclusion criteria were pediatric patients aged 0 to 21 years admitted to Shriners Hospital for Children-Boston from January 1, 2010 to January 1, 2021 for burn injury, who subsequently required follow-up complex ear reconstruction for ear deformities after burn injury. Of these patients, those with lop ear deformities who required construction were examined. Using the Trauma Registry of the American College of Surgeons (TRACS) and Shriners Hospital for Children Information Source (SHCIS) databases, we identified 12 eligible patients. Surgical procedure data was obtained from the SHCIS electronic medical records. Surgical photographs were obtained with patient informed consent. Those patients who required Medpor or cartilage frame reconstructions were excluded.

**Results:**

12 patients required an otoplasty-type procedure to obtain desired results or symmetry with the contralateral ear. This technique involved a posterior incision in the auriculomastoidmastoid sulcus, are you a year and cartilage modification with sutures to to re-create the antihelical fold an superior crus as well as conchal setback sutures.

**Conclusions:**

Because ears are bilateral structures, symmetry is extremely poor. Because of multiple contractile forces on the external ear from secondary healing or contraction of skin grafts, the ear is often displaced anteriorly and the upper portions become folded. Treatment requires reconstruction of the natural folds of the ear and shortening of the helical rim to mastoid distance. this requires an otoplasty approach with a posterior incision,, exposure of the cartilaginous framework and modification of the cartilage with incisions and sutures. This technique however requires adequate soft tissue coverage of the ear.

